# Supplementary material for: Population receptive field estimates for motion-defined stimuli
Source: Neuroimage. 2019 Oct 1;199:245–60. doi: 10.1016/j.neuroimage.2019.05.068 (PMC6693563; doi:10.1016/j.neuroimage.2019.05.068)
Supplement: Supplementary document — containing an example delineation of TO1 and TO2, eccentricity plots and analyses using a R2 threshold of 0.1. [file mmc1.docx]

Population receptive field estimates for motion-defined stimuli

Anna E. Hughes ^1,2*^, John A. Greenwood^1^, Nonie J. Finlayson^1,3^ and D. Samuel Schwarzkopf^1,4^

**Supplementary material**

**Delineation of TO1 and TO2**

**
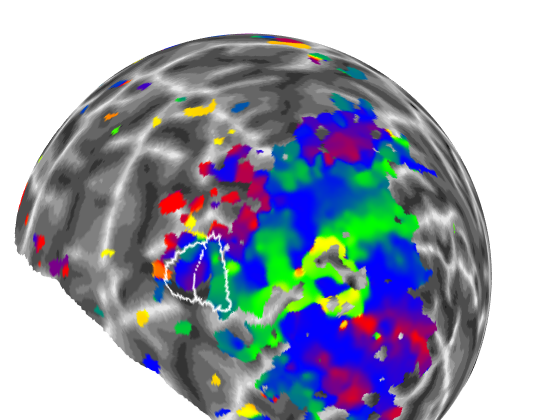
**

**Figure S1:** An example delineation of areas TO1 and TO2 on the smoothed retinotopic maps for a single participant (using data from the dot-only bars in Experiment 1, and a threshold of 0.06). The border between them was determined as being an upper vertical meridian reversal (red), and the outer borders on either side were either a horizontal (blue) to lower vertical meridian (green). For analyses however, we combine TO1 and TO2 into one MT+, so the exact distinction is not relevant for analysis.

**Plots of pRF size against eccentricity**


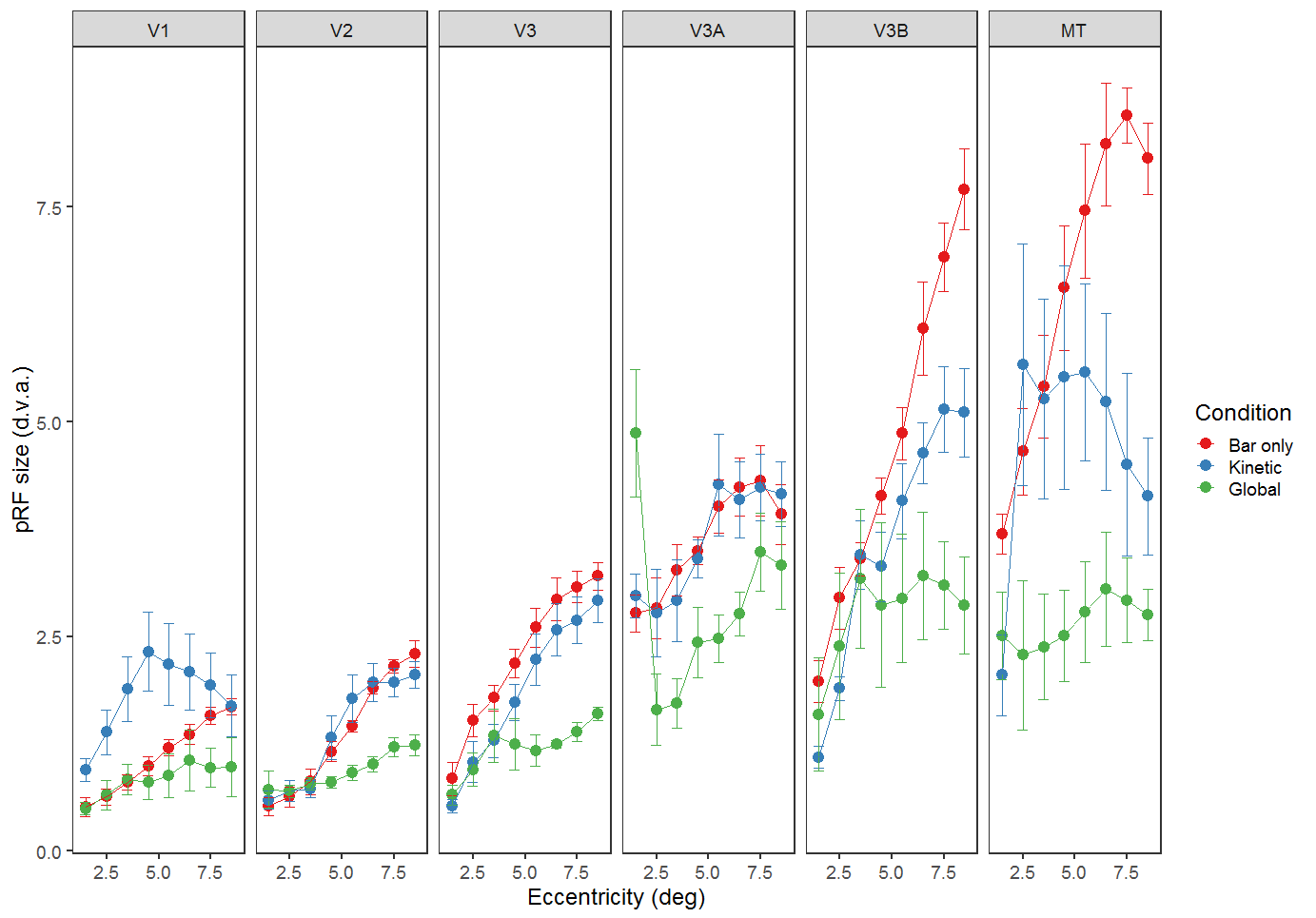


**Figure S2:** pRF size plotted against eccentricity for the different experimental conditions and brain regions in Experiment 1.


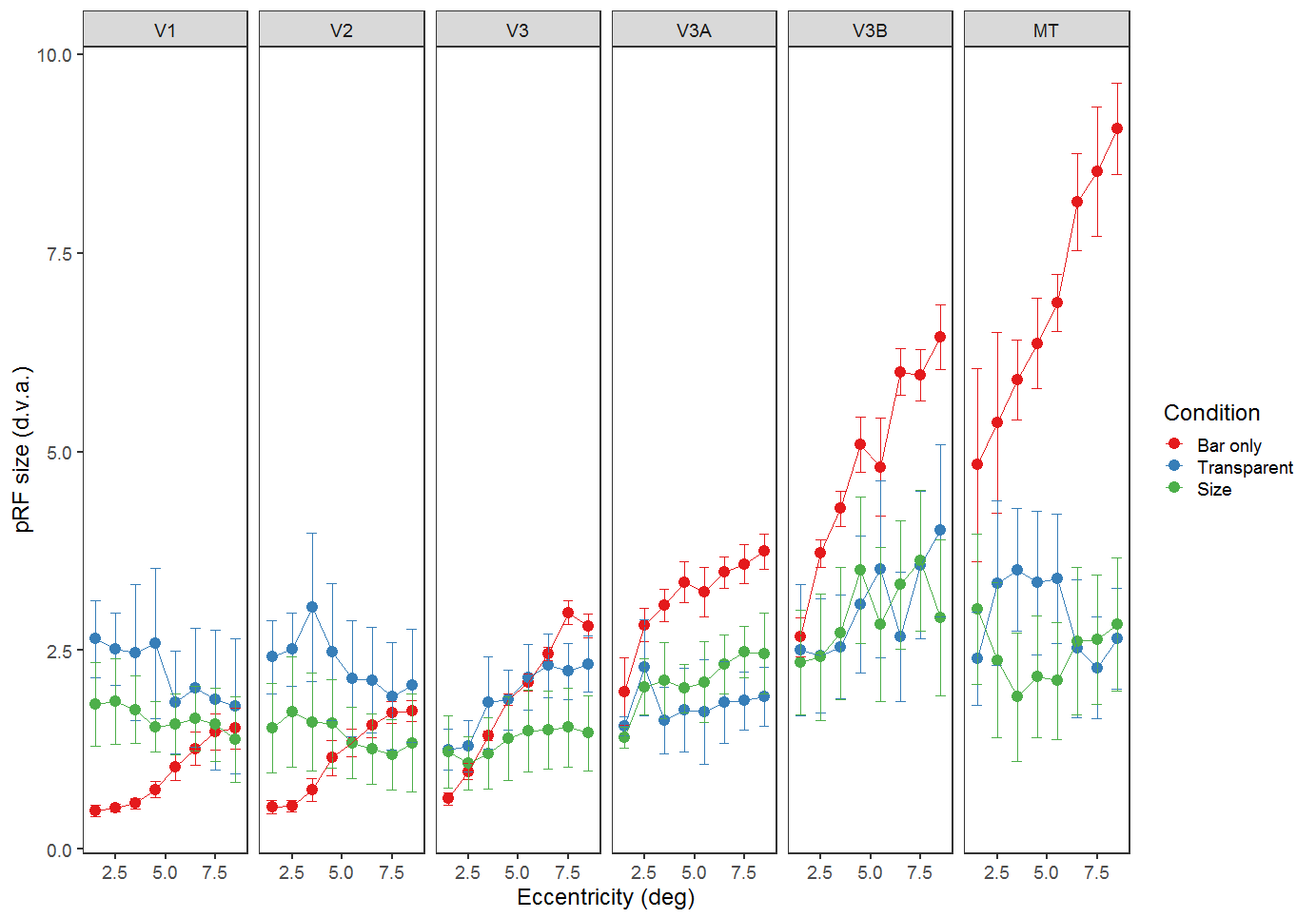


**Figure S3:** pRF size plotted against eccentricity for the different experimental conditions and brain regions in Experiment 2.

**Analyses using an R^2^ threshold of 0.1**

As our experimental conditions often showed relatively weak and sparse responses, we used a fairly liberal R^2^ threshold of 0.05 in the main analyses. Here, we show the same analyses using a more conservative R^2^ threshold of 0.1. Overall, the results are highly comparable. We did not conduct formal statistical analysis due to the relatively high levels of missing data in some conditions and for some participants.

*Experiment 1*


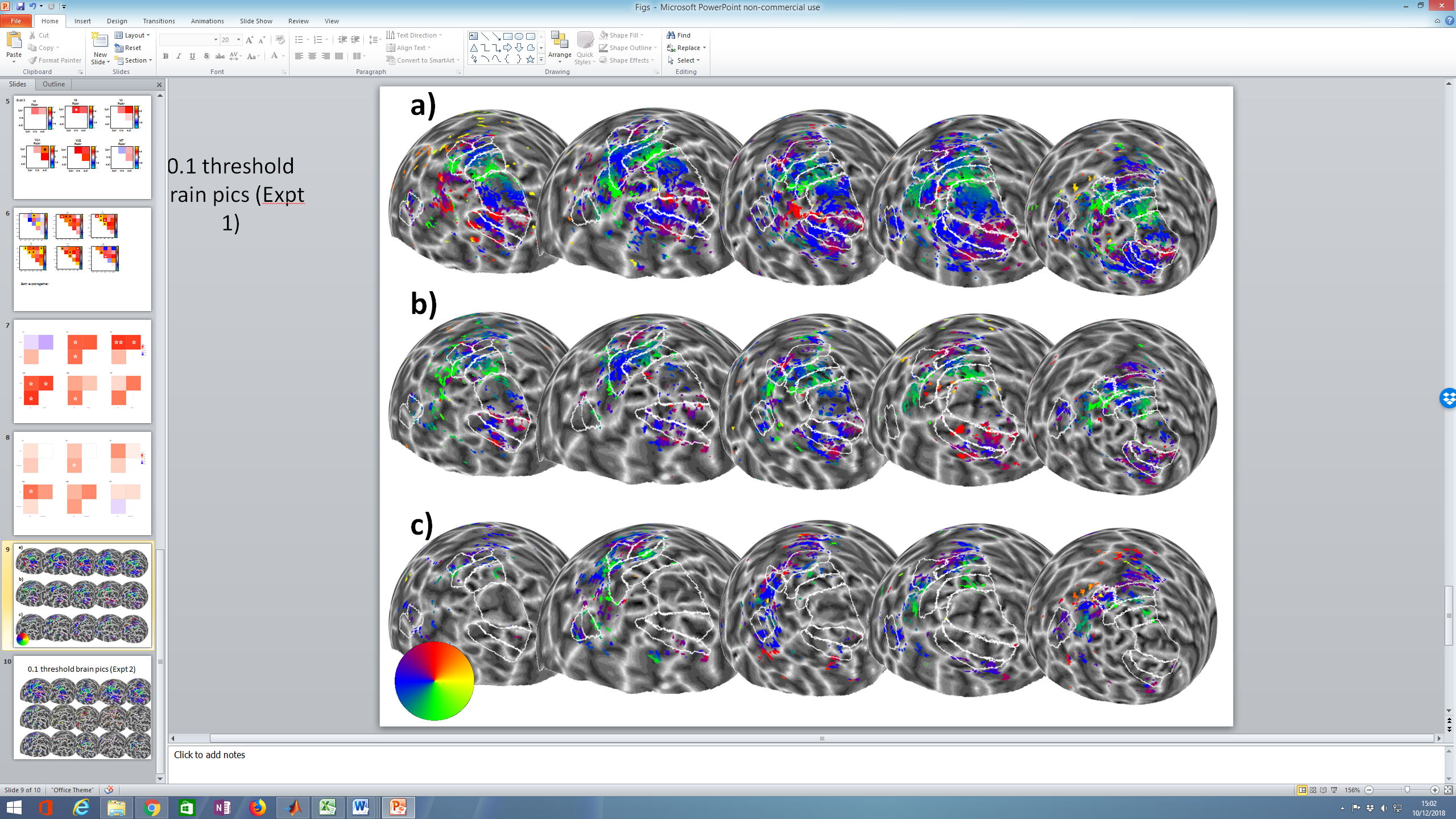


**Figure S4.** Sphere projection of polar angle data for the left hemispheres of all participants in Experiment 1 using an R^2^ threshold of 0.1. The colour of each vertex indicates the polar angle for the corresponding pRF centre (as indicated by the colour wheel). Each person’s data forms a column (subject 1 is on the far left, and subject 5 is on the far right), and stimulus condition forms a row. Manual delineations of visual areas V1, V2, V3, V3A, V3B and hMT+ (TO1/2) are shown. (**a**) Polar angle estimates for the ‘bar-only’ stimulus condition. (**b**) Polar angle estimates for the ‘kinetic’ stimulus condition. **(c)** Polar angle estimates for the ‘global’ stimulus condition.


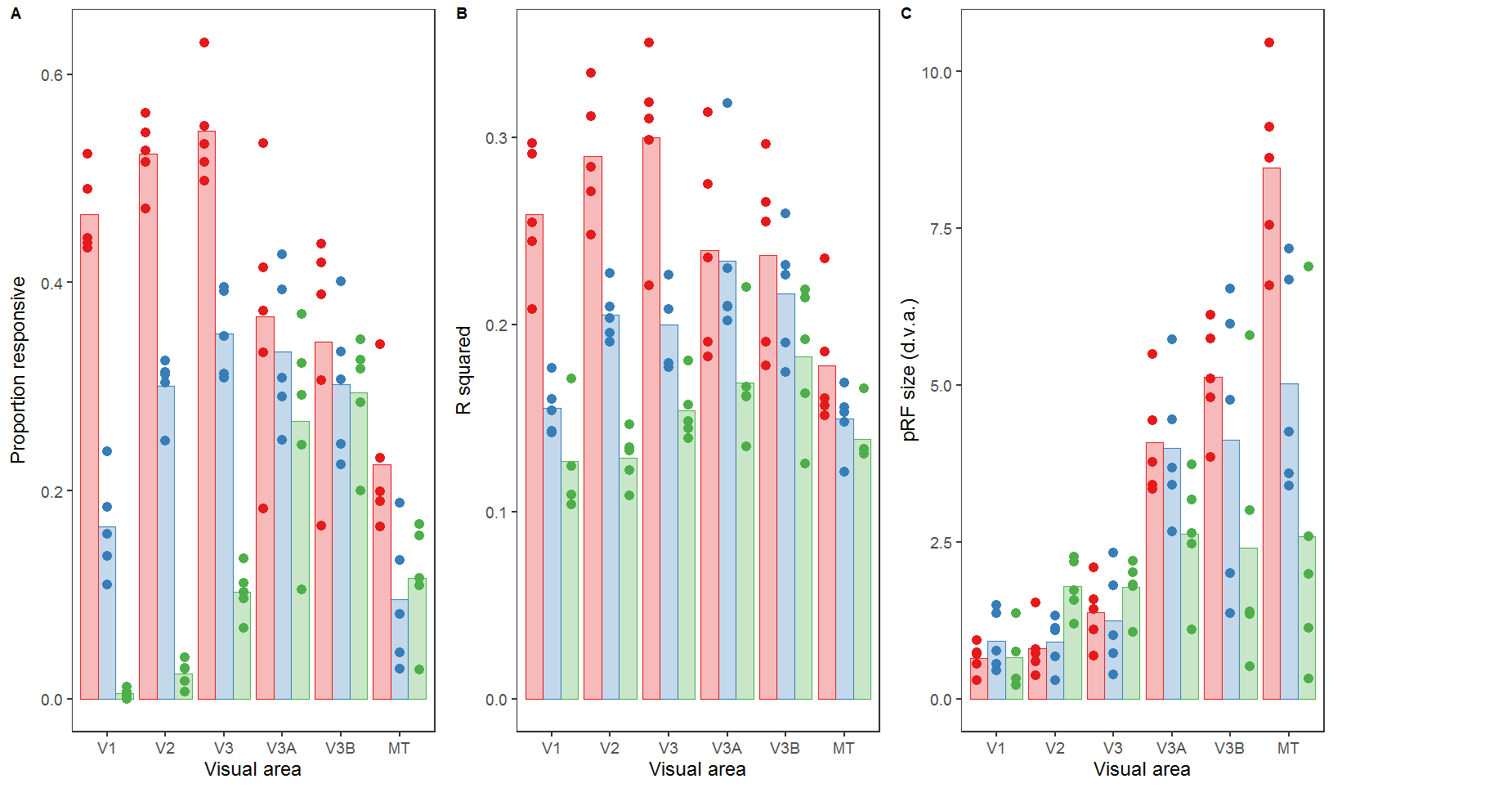


**Figure S5.** **(a)** Proportion of vertices responding, **(b)** goodness-of-fit and **(c)** pRF sizes for each condition and visual area in Experiment 1 with an R^2^ threshold of 0.1. The bars show the mean values across all subjects, and the points are individual data for each subject. Panel (a) plots the mean proportion of vertices responding for each subject, whereas (b) and (c) plot the median goodness-of-fit values and pRF sizes respectively. Subject 2 is missing data for the V1 global condition.

*Experiment 2*


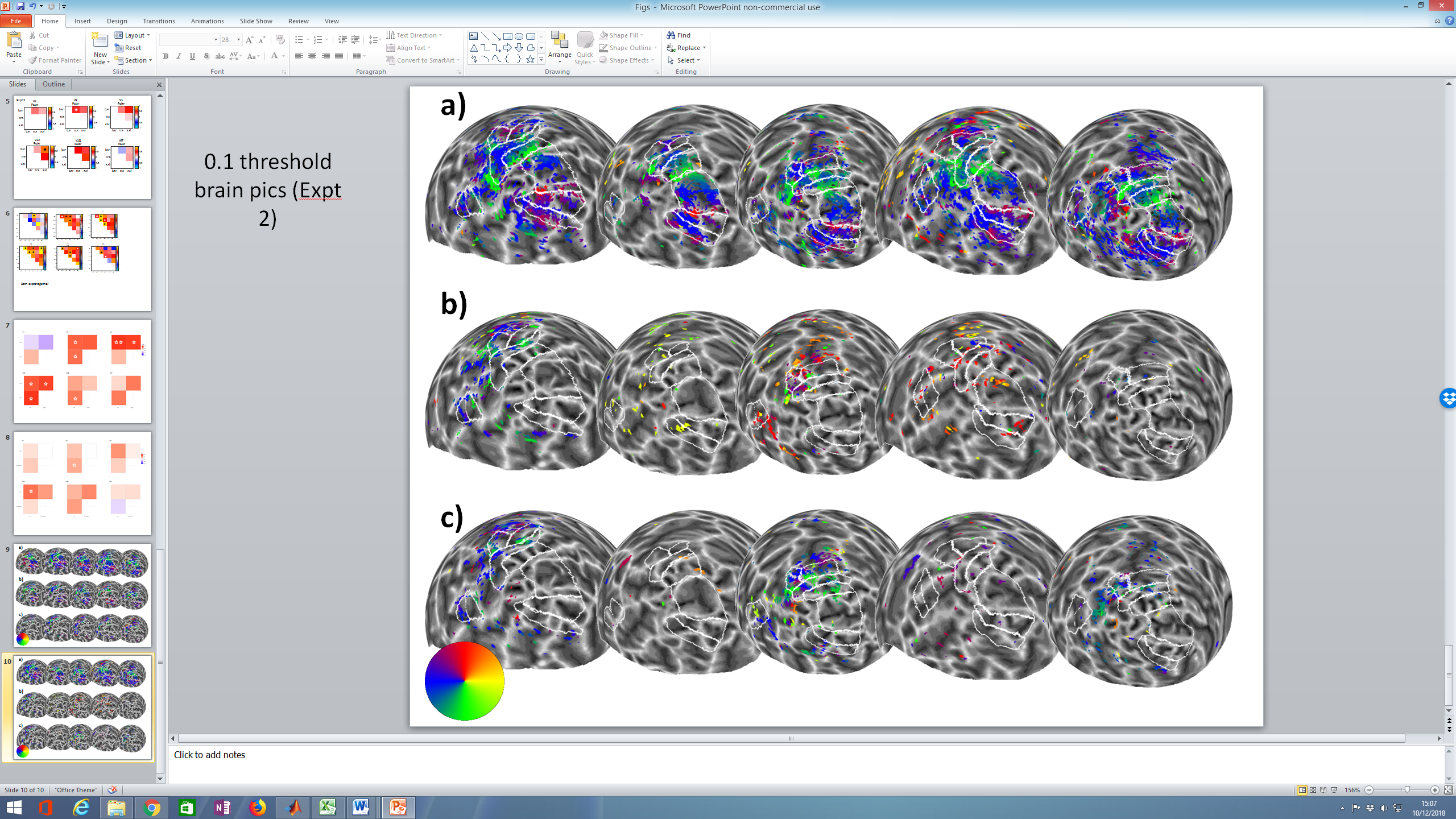


**Figure S7.** Sphere projection of polar angle data for the left hemispheres of all participants in Experiment 2 with an R^2^ threshold of 0.1. The colour of each vertex indicates the polar angle for the corresponding pRF centre (as indicated by the colour wheel). Each person’s data forms a column (subject 1 is on the far left and subject 5 is on the far right), and stimulus condition forms a row. Manual delineations of visual areas V1, V2, V3, V3A, V3B, and hMT+ (TO1/2) are shown (if the subject had taken part in Experiment 1, the delineations from this experiment were used). (**a**) Polar angle estimates for the ‘bar-only’ stimulus condition. (b) Polar angle estimates for the ‘transparent’ stimulus condition. (**c**) Polar angle estimates for the ‘size-defined’ stimulus condition.


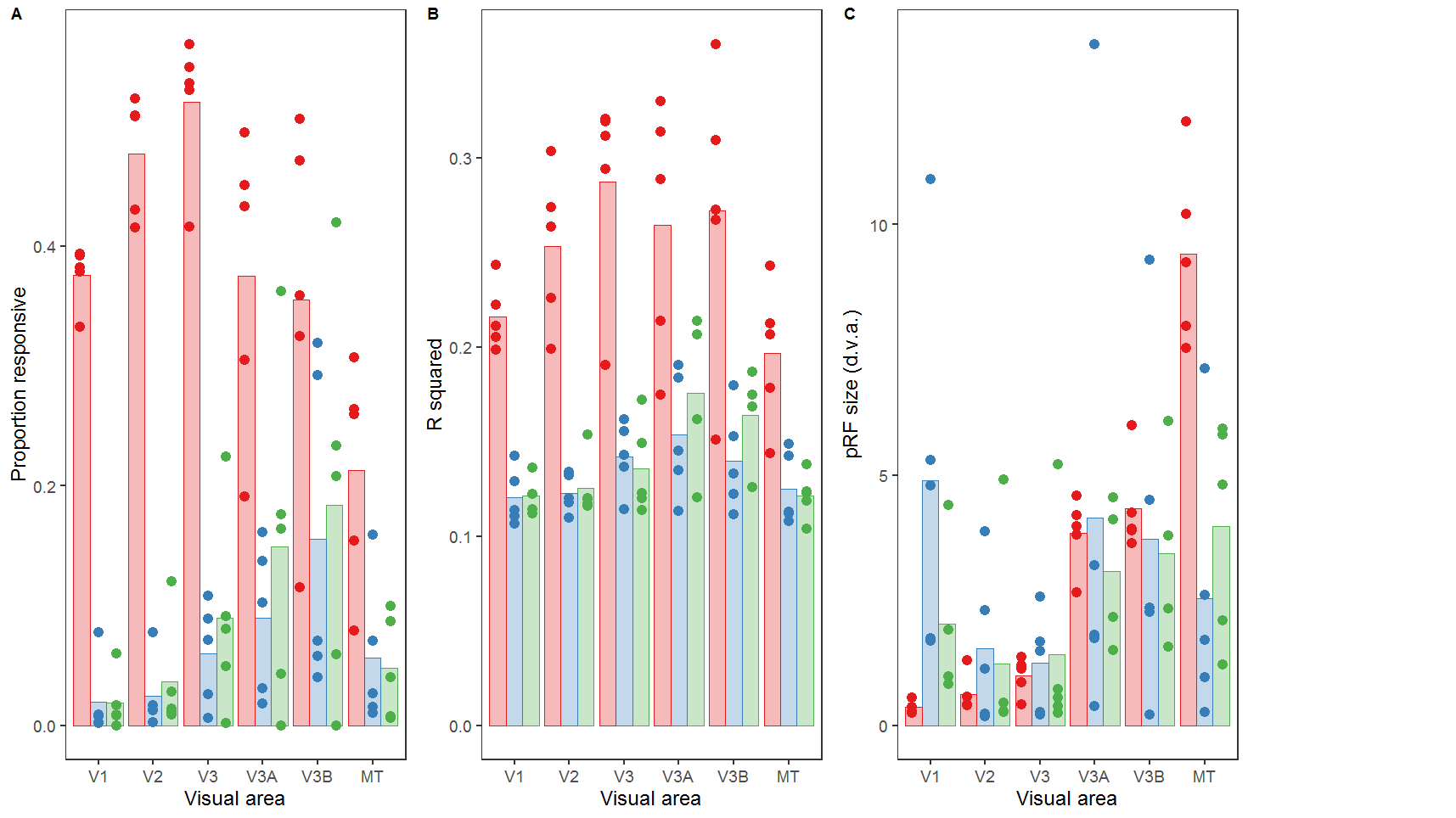


**Figure S8.** **(a)** Proportion of vertices responding, **(b)** goodness-of-fit and **(c)** pRF sizes for each condition (bar-only, transparent, and size-defined) and visual area in Experiment 2 with an R^2^ threshold of 0.1. The bars show the mean values across all subjects, and the points are individual data for each subject. In (a), this is the mean proportion of vertices responding for each subject, whereas for (b) and (c) these are the median goodness-of-fit values and pRF sizes respectively. Subject 2 is missing data for the size condition in V1, V3A and V3B.
