# Supplementary figures and images for: Population receptive field estimates for motion-defined stimuli
Source: Neuroimage. 2019 Oct 1;199:245–60. doi: 10.1016/j.neuroimage.2019.05.068 (PMC6693563; doi:10.1016/j.neuroimage.2019.05.068)

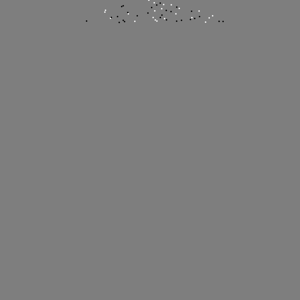

Supplement: Video S1 — Example bar-only stimulus. [file mmc3.gif]

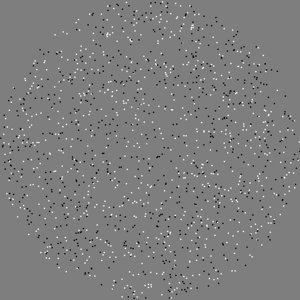

Supplement: Video S2 — Example kinetic stimulus. [file mmc4.gif]

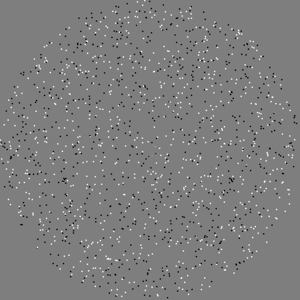

Supplement: Video S3 — Example global stimulus. [file mmc5.gif]

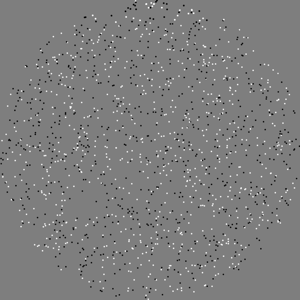

Supplement: Video S4 — Example transparent bar-only stimulus. [file mmc6.gif]

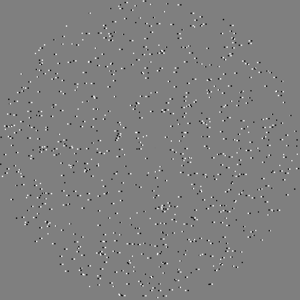

Supplement: Video S5 — Example transparent motion stimulus. [file mmc7.gif]

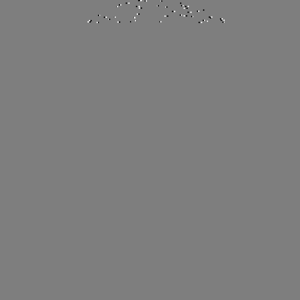

Supplement: Video S6 — Example size-defined stimulus. [file mmc8.gif]
